# Exploring Retirement Concerns of Basic School Teachers in Ghana

**DOI:** 10.1155/jare/3735264

**Published:** 2026-07-29

**Authors:** John N-yelbi, Dandy George Dampson, Matthew Kojo Namale

**Affiliations:** ^1^ Department of Counselling Psychology, Faculty of Applied Behavioural Sciences in Education, University of Education, Winneba, Ghana, uew.edu.gh; ^2^ Department of Educational Foundations, School of Education and Life-Long Learning, University of Education, Winneba, Ghana, uew.edu.gh

**Keywords:** basic school, concerns, pre-retirement planning, retirement, teachers

## Abstract

The study explored the retirement concerns of basic school teachers in Ghana. It employed a qualitative phenomenological research design. 24 teachers aged 50–55, who were in the pre‐retirement stage and approaching statutory retirement of 60, were sampled using the maximum variation sampling technique and interviewed. In‐depth semistructured interviews were used to gather data, which were then subjected to thematic analysis. The findings revealed financial concerns, healthcare issues and social isolation as retirement concerns. The study found that teachers’ retirement concerns were centred around three main categories: psychological concerns resulting from anxieties and uncertainties about life after active employment, social concerns about evolving family and social relationships, and financial concerns about financial insecurity in retirement. The study recommends that Ghana Education Service collaborates with financial institutions to enhance teachers’ budgeting, pension and savings skills. It suggests organising financial literacy programmes to promote savings and investment diversification. It recommends pre‐retirement programmes to improve their emotional preparedness for retirement.

## 1. Introduction

Retirement represents a pivotal life transition, marking the formal cessation of professional activity and the entry into a distinct stage of social and personal identity often associated with advancing age [1]. While a plethora of studies offer a broad understanding of retirement concerns, there remains a critical need for context‐specific insights into the retirement concerns of basic school teachers in Ghana. Much of the global literature is situated within western, developed contexts, which may not adequately reflect the socioeconomic, cultural and institutional realities of African settings. In Ghana, for instance, retirees often contend with bureaucratic pension systems, limited access to healthcare and weakened family support structures. This study seeks to address these contextual gaps by generating contextually grounded evidence essential for designing culturally relevant and effective interventions.

However, viewing this phase merely as an end to work overlooks its potential as an opportunity for personal growth, the exploration of new interests and engagement in part‐time occupations or volunteer activities. This perspective reflects a shift from complete work cessation to a more flexible transition into later‐life activities [1]. The concept of retirement concerns encompasses individuals’ views, expectations and emotional responses regarding this significant life phase. These concerns are influenced by a complex interplay of factors, including personal circumstances, financial preparedness, emotional preparedness, transformations in identity and prevailing social norms [2].

Research consistently indicates that individuals’ concern about retirement can profoundly affect their psychological well‐being. For instance, positive perspectives on retirement—such as viewing it as a time for leisure, personal development and a significant life transition—are associated with enhanced mental health outcomes [2]. Conversely, negative retirement concerns, often rooted in fears of social isolation or a perceived loss of purpose, can lead to increased mental health problems [3, 4]. For individuals such as teachers whose professional identity is deeply intertwined with their work, such as teachers, retirement can represent a significant psychological and social recalibration. The profession of teaching often provides a strong sense of purpose, daily social interaction and a community of colleagues, making the transition out of this role a profound rupture for some.

Retirement concerns comprise the financial, social, psychological and health‐related worries or apprehensions experienced by individuals regarding their preparedness for retirement, adjustment to postemployment life and ability to maintain well‐being and economic security after exiting the workforce [5]. Teachers’ concerns about retirement are particularly critical as they reflect their unique experiences and expectations surrounding this transition. Studies have shown that the calibrations can significantly impact teachers’ overall quality of life during retirement [6]. Furthermore, Osuji [7] emphasises that teachers’ beliefs and attitudes about retirement programmes shape their outlook on this phase of life. Teachers approaching retirement may engage in negative cognitive assessments, perceiving retirement as a threat rather than an opportunity for social engagement and psychological development. Such negative impressions contribute to heightened stress levels that can adversely affect overall psychological well‐being [8].

Several factors consistently emerge as crucial determinants of retirement concerns. Research indicates that individuals possessing higher financial literacy tend to hold more favourable aspirations for retirement [9]. Studies also confirm that those with clearer retirement goals and greater financial literacy are more inclined to save and engage in proactive financial planning [10]. Similarly, lack of financial knowledge often leads to associations of retirement with uncertainty and instability, fostering increased anxiety about the future. However, Kim and Moen [4] found a positive correlation between individuals’ concerns about their postretirement income sources, such as pensions and savings, and their overall psychological well‐being. Many teachers are not financially prepared for retirement as documented by Sterns and Spokus [11] study, which reported feeling unprepared for managing postretirement finances due to insufficient knowledge about pension plans and savings strategies.

There is evidence that many people overlook concerns about their long‐term financial security, such as retirement well‐being, when faced with challenging financial situations [12]. Financial limitations make it difficult for people to think about less pressing issues like retirement [13]. This study articulates its distinctive contribution by highlighting the potential to advance theoretical understanding and inform practice regarding teachers’ retirement concerns, particularly their inclination towards insufficient savings. Due to their inability to save for retirement, teachers’ subjective well‐being and mental health are negatively impacted by financial difficulties [14].

Healthcare costs also play a significant role in shaping retirement perspectives. As individuals age, concerns about medical expenses become increasingly prominent. Goldman and Smith [15] observed that workers anticipating substantial healthcare costs in retirement are more likely to feel apprehensive about their future, particularly in systems lacking comprehensive healthcare coverage. This apprehension can foster negative attitudes towards retirement; with many fearing, they will be unable to afford necessary healthcare cost after retirement.

Social isolation represents another critical factor influencing retirement concerns. Substantial research evidence reported that individuals with strong social ties tend to have more positive attitudes about retirement [16, 17]. Relatedly, a study by Shin et al. [18] found that individuals derive a substantial portion of their identity and social connections from their work. Thus, the transition into retirement can result in feelings of loneliness and disconnection from established social networks. Maintaining community engagement and social connections postretirement is essential for mitigating feelings of isolation and enhancing overall life satisfaction during the retirement phase [19, 20].

In Ghana, more than 1.6 million active workers including basic school teachers contribute to the Social Security and National Insurance Trust, a governmental pension plan [21]. Defined benefit and defined contribution pension plans are included in the three‐tiered Social Security and National Insurance Trust. Employees must contribute 13.5% and 5% of their income, respectively, to the first and second tiers [21]. According to Ongoh et al. [22], the majority of the pensioners did not seek to improve their interpersonal relationships (59.9%), social support (73.2%) or social networks (65.9%).

### 1.1. Research Questions

1. What concerns do basic school teachers in Nanumba North, Jasikan and Effutu Municipalities have about retirement?
2. How does pre‐retirement planning shape basic school teachers’ concern for retirement in Nanumba North, Jasikan and Effutu Municipalities?

### 1.2. Theoretical Review

The theoretical framework for this study is anchored in three core theories: life‐course perspective, stress and coping theory and social identity theory. Together, these theories provide a robust framework for understanding teachers’ retirement concerns and the influence of pre‐retirement programmes. These theoretical reviews help to explain the complex interplay of individual experiences, psychological processes and broader social and structural factors that shape the retirement transition.

### 1.3. A Life‐Course Perspective

The theory serves as a foundational lens, positioning retirement as a dynamic, evolving process within an individual’s entire life trajectory, rather than a singular event [23]. This perspective emphasises how cumulative experiences, transitions and resources accumulated throughout life, such as financial literacy and social capital, shape an individual’s preparedness for and adaptation to retirement. It recognises that retirement is not a uniform experience but is influenced by an individual’s unique biography and the societal contexts in which they live.

### 1.4. Stress and Coping Theory

This theory provides a framework for understanding the psychological dimensions of retirement. When retirement is perceived negatively, particularly as a ‘threat’ rather than an ‘opportunity’, it can induce significant stress and apprehension [24]. This theory helps to explain how pre‐retirement planning and counselling programmes can function as crucial coping resources. By providing information, support and strategies, these programmes can enhance individuals’ perceived control over the transition, thereby reducing anxiety and fostering a more adaptive response. The provision of knowledge, particularly in areas like financial literacy, can have a profound psychological impact, reduce anxiety and increase perceived control by demystifying complex financial and health landscapes. This highlights how psychoeducation acts as a crucial bridge between practical planning and psychological well‐being.

### 1.5. Social Identity Theory

This is particularly relevant given the strong professional identity often associated with teaching. Work, especially in a collaborative environment like a school, frequently provides a significant source of social identity, belonging and daily interaction [25]. Retirement can challenge this deeply ingrained identity, leading to feelings of loneliness and disconnection from established social networks. This framework helps to explore how social integration initiatives within pre‐retirement programmes aim to facilitate the formation of new social identities and networks, helping individuals to maintain a sense of belonging and purpose beyond their professional roles.

Collectively, these theoretical perspectives inform the study’s investigation into the complex interplay of individual concerns, psychological responses and systemic factors, as well as the transformative potential of targeted pre‐retirement interventions for teachers. The study recognises that teachers’ retirement concerns are not solely a matter of individual psychological disposition or planning; they are profoundly shaped by the socioeconomic and institutional realities of Ghana, such as the specific healthcare system and family support structures. This necessitates an explicit acknowledgement of the interplay between individual agency and structural constraints, reinforcing the argument for systemic interventions alongside individual support.

## 2. Methodology

This study adopted an interpretive paradigm to understand the subjective meaning and experiences of teachers as they navigate their concerns about retirement. This approach facilitated a comprehensive understanding of the factors that shape teachers’ views on retirement. These include financial concerns, healthcare concerns and social integration [26] by utilising a qualitative phenomenological research design. The study aimed to uncover the essence of teachers’ experiences and the meanings they attribute to their impending retirement. This research design focuses on individuals’ subjective interpretations and experiences of the world, providing in‐depth and detailed information, allowing for flexible data collection methods [27].

The study was conducted in three municipalities: Nanumba North (Northern Region), Jasikan (Oti Region) and Effutu (Central Region). These sites were deliberately selected using a maximum variation sampling strategy to capture diverse socioeconomic, cultural and institutional contexts that shape retirement concerns among basic school teachers in Ghana. Nanumba North represents a predominantly rural setting with limited access to formal financial and healthcare services, highlighting structural vulnerabilities in retirement preparedness. Jasikan, located in a semiurban area, reflects transitional dynamics where teachers balance traditional communal obligations with emerging financial literacy practices. Effutu, a coastal urban municipality, offers relatively better access to pension services and healthcare but presents challenges related to social identity and isolation in retirement. By including municipalities from northern, middle and coastal Ghana, the study ensured regional diversity and enhanced the transferability of findings. This selection strategy strengthens methodological rigour by situating retirement concerns within varied socioeconomic and cultural realities, thereby providing a comprehensive understanding of the phenomenon.

A total of 24 teachers were selected using maximum variation sampling to ensure representation across key demographic variables that influence retirement concerns. This included age, gender, years of service and proximity to retirement. Age captures life‐stage differences in preparedness; gender reflects social roles and expectations; years of service determine pension entitlements and career trajectories; and proximity to retirement highlights the immediacy of concerns. By deliberately including teachers across these categories, the study sought to generate findings that reflect both commonalities and contrasts in retirement anxieties, thereby enhancing validity and transferability.

To capture the diversity of retirement concerns among basic school teachers in Ghana, the researchers employed the maximum variation sampling technique. This approach was used to guarantee coverage across important demographic factors that influence retirement experiences, such as age, gender, years of service and retirement proximity. The factors selected are significant as they affect both the structural and psychological dimensions of retirement. Specifically, years of service influence pension entitlements and career trajectories; gender highlights the impact of social roles and expectations; age indicates variations in retirement readiness linked to different life stages; and proximity to retirement underscores the urgency of concerns. To enhance the validity and transferability of the findings, the study intentionally included teachers from all these categories, ensuring a dataset that reflects both commonalities and differences in retirement concerns. This technique further enhanced the richness of the qualitative data collected, allowing for a more comprehensive understanding of the diverse experiences within the target population. The 24 participants were selected based on data saturation, and it was deemed to be a reasonable number for a qualitative phenomenological study, allowing for in‐depth exploration without becoming unmanageable [28, 29].

Data collection continued until data saturation was reached. Throughout the interview process, saturation was monitored by closely observing the emergence of new themes. The research team noticed that after about the twentieth interview, participants were reiterating concerns that had already been noted, like social isolation, healthcare expenses and financial instability, without offering new insights. Four further interviews were done to ensure rigour, but they did not produce any new categories or insights beyond what was already known. The dataset had reached thematic redundancy at this point; therefore, the researchers concluded that no more data collection was required. This choice confirms that the sample sufficiently reflected the variety of concerns pertinent to the study’s goals by reflecting the idea that saturation happens when further interviews fail to provide fresh information. This decision aligns with Vasileious et al. [29], who suggest that a thematic analysis sample of 5–25 participants is adequate. Qualitative research prioritises rich understanding, and by purposefully selecting participants, the researcher fostered strong relationships, resulting in more detailed data and improved findings quality.

Semistructured interview guide was used to collect data from the target population. The use of semistructured interview was well‐justified for a phenomenological study, as it allowed participants to express their thoughts and feelings freely, while also guiding the conversation to cover specific thematic areas relevant to the research questions. Each interview lasted approximately 60–90 minutes and was conducted in a comfortable setting chosen by the participants to encourage openness and rapport. All interviews were audio‐recorded with participants’ consent and subsequently transcribed verbatim for analysis, ensuring accuracy and completeness of the raw data.

### 2.1. Ensuring Trustworthiness and Triangulation

Several rigour‐enhancing techniques improved the qualitative interviews’ credibility. First, member checking was used to increase credibility by sharing interview summaries and preliminary interpretations with participants to confirm the veracity of their reports and the researchers’ interpretations. In order to promote reflexivity and analytical rigour, peer debriefing was also carried out through frequent conversations with independent qualitative researchers who explored emergent themes, questioned presumptions and offered alternative interpretations. Multiple coders were involved in the analytic process to ensure intercoder agreement. Transcripts were independently classified and compared, and any inconsistencies were discussed and resolved to improve consistency and dependability. Methodological and analytical techniques were used to accomplish triangulation. Analyst triangulation was bolstered by the participation of various researchers in coding and interpretation, while data triangulation was maintained by comparing opinions across individuals with different backgrounds and experiences. When combined, these strategies improved the findings’ dependability, trustworthiness and confirmability, guaranteeing that the outcomes were solid and supported by evidence.

### 2.2. Data Analysis

Braun and Clarke’s [30] reflexive thematic analysis was used to analyse the data, utilising a hybrid coding strategy that included deductive and inductive techniques. While inductive coding allowed new, context‐specific themes to arise immediately from participant narratives, deductive coding was guided by sensitising notions from stress and coping theory, social identity theory and life‐course perspective. This combination improved conceptual rigour and empirical richness by ensuring that conclusions were both theory‐informed and based on the real‐world experiences of Ghanaian basic school teachers. By combining both, the analysis ensured that findings were simultaneously theory‐informed and data‐driven, enhancing credibility, transferability and scholarly impact. This hybrid approach was chosen because it balances theoretical rigour with empirical openness. Solely deductive coding risked imposing pre‐existing frameworks onto the data, potentially overlooking context‐specific concerns such as healthcare costs under Ghana’s ‘cash and carry’ system. Conversely, purely inductive coding might have produced rich descriptive themes but lacked theoretical integration, limiting the study’s contribution to broader gerontological discourse. By combining both, the analysis ensured that findings were simultaneously theory‐informed and data‐driven, enhancing credibility, transferability and scholarly impact.

In order to create meaning categories or patterns, themes and subthemes, it was necessary to sort through, compile or combine codes. In order to make sure that all coded data were included in the themes and that the themes generally accurately represented the data, the following stage of the analysis involved reviewing and refining the preliminary themes with respect to the research goal and questions. Lastly, the study’s major themes and subthemes were identified and presented. The data analysis procedure is shown in Table 1. It demonstrates how the themes were named after the data were coded.

**TABLE 1:** Demographic characteristics of participants (*n* = 24).

Variable	Category	Number of participants
Age	30–39 years	6
	40–49 years	8
	50–55 years	10

Gender	Male	15
	Female	9

Years of service	< 10 years	5
	10–20 years	9
	20+ years	10

Proximity to retirement	< 5 years	8
	5–10 years	10
	> 10 years	6

Throughout this process, attention was paid to ensuring that participants’ voices were accurately represented, maintaining the integrity of their experiences while drawing meaningful conclusions from the data. This rigorous process enhanced the transparency and credibility of the study’s analytical approach. In interpretive research, the researcher is the primary instrument, and while systematic steps were followed, self‐awareness regarding potential influences on data interpretation is crucial for methodological rigour. As presented in Table 2, three major themes, financial concerns, healthcare expenses and social isolation, emerged from the thematic analysis as the retirement concerns of basic school teachers. Furthermore, three overarching themes emerged from the thematic analysis of how pre‐retirement planning influences retirement concerns among basic school teachers. These themes included financial planning, social planning and psychological planning, each representing distinct preparatory strategies identified by participants, as shown in Table 3.

**TABLE 2:** The process of data analysis of basic school teachers’ retirement concerns in Ghana.

Main theme	Subthemes
Financial concerns	Retirement often precipitates financial strain, encompassing insecurity about income sources, inadequate savings, unforeseen expenses, rising medical and utility costs, family obligations and diminished earning opportunities.
Healthcare expenses	Teachers preparing for retirement often foresee substantial health‐related challenges, including escalating medical costs, limited access to care, unmet long‐term care needs, chronic health conditions, gaps in national insurance coverage and reliance on out‐of‐pocket payment systems.
Social isolation	Retirement can intensify social challenges, including fear of losing connections, diminished workplace ties, limited community engagement and feelings of loneliness, dejection or abandonment, underscoring the importance of strategies to sustain meaningful relationships.

**TABLE 3:** The process of data analysis of how pre‐retirement planning shape retirement concerns of basic school teachers.

Main theme	Subthemes
Financial planning	Seek financial education, participate in financial literacy planning workshops and programmes.
Social planning	Social support systems, connections with coworkers, friends, family members and loved ones.
Psychological planning	Emotional well‐being sensitisation, psychological awareness training and stress management programmes.

### 2.3. Ethical Considerations

In order to adhere to ethical standards, participants volunteered to participate in the study after being informed of its objective and what was expected of them. The participants’ privacy and confidentiality were protected by not disclosing their names and identities during data collection, analysis and reporting of the study results. The privacy and confidentiality of the interview environment were very carefully ensured during the interview, data analysis and dissemination of the findings. Data were stripped of identifying characteristics by providing a code to specific information [31]. To ensure this in this study, the genuine identity of the research participants was masked by providing them with a pseudonym and changing other identifying characteristics that may make it difficult for participants to be identified. The study received Institutional Review Board (IRB) approval, a crucial requirement for research involving human subjects.

## 3. Results

The empirical results derived from the qualitative study address the research questions, revealing key concerns of retirement among basic school teachers in Ghana and how pre‐retirement planning shapes these concerns. The findings are organised under clear headings corresponding to the main themes and subthemes and supported by illustrative participant quotes.

### 3.1. Basic School Teachers’ Retirement Concerns

The first research question explored how basic school teachers perceive retirement, particularly regarding financial preparedness, healthcare concerns and social integration. The analysis revealed that teachers’ retirement concerns are closely linked to significant financial concerns, anticipated healthcare expenses and fears of social isolation. Table 1 provides a summary of the primary themes and subthemes that emerged from the study regarding these concerns.

### 3.2. Financial Concerns

Teachers expressed significant anxiety regarding financial inadequacy during retirement, a predominant theme that emerged from the data. This included worries about insufficient postretirement income, a lack of adequate savings and the burden of unforeseen financial commitments, particularly concerning dependents’ education and daily living expenses.

One participant articulated this burden, stating:

“*With three children in school and a non-working spouse, I rely solely on my monthly salary, which is insufficient to cover educational expenses*.” (Participant 3)

Another teacher echoed this sentiment, highlighting the challenge of living on a fixed, meagre income postretirement, especially when supporting children in higher education and managing existing loans.

“*What if unexpected expenses arise? As I consider retirement, I can’t help but wonder about unforeseen financial challenges that may arise. The cost of living is very high these days*.” (Participant 5)

Retirement concerns among basic school teachers in Ghana are complex, influenced by financial insecurity, healthcare fears and social isolation. Stress and coping theory illustrate these concerns as continuous stressors, while the life‐course perspective shows retirement as a result of long‐term disadvantages. Social identity theory indicates that retirement threatens professional identity and social connections, heightening fears of isolation. Therefore, retirement preparedness should include financial literacy, healthcare reforms and community engagement strategies, moving beyond mere financial planning to address these interconnected challenges in vulnerable contexts.

### 3.3. Healthcare Expenses

Concerns about anticipated medical costs were another prominent theme. A good number of participants admitted to having underlying health conditions requiring monthly medical attention, which incur substantial expenses. A teacher from Jasikan Municipality shared,

*“Unexpected medical expenses, often not covered by national insurance, create financial uncertainty and emotional distress*.” (Participant 1)

Another participant highlighted the ongoing financial drain from a past accident, stating:

“*Half of my salary goes to medical expenses after an accident, leaving me under constant financial pressure*.” (Participant 12)

This narrative illustrates how retirement concerns are complex and multifaceted, involving identity issues, accumulated structural disadvantages and stress reactions. It emphasises how urgently policy changes that improve healthcare coverage, increase pension protections and promote identity continuity for teachers nearing retirement are needed.

### 3.4. Social Isolation

The fear of losing social connections emerged as a critical factor influencing retirement concerns. Teachers expressed apprehension about missing the camaraderie of colleagues and students, which had been a constant source of daily social interaction. One participant from Effutu Municipality reflected:

“*Retirement means losing the daily camaraderie and community of the staffroom.*” (Participant 20)

Another teacher from Nanumba North Municipality shared a similar viewpoint, stating:

*“Retirement threatens the daily social interaction with students and colleagues, raising fears of isolation.”* (Participant 18)

This narrative illustrates how retirement concerns extend beyond loss of identity and social affiliation, in addition to financial insecurity. In order to ensure that teachers keep both identity continuity and emotional well‐being after leaving employment, it emphasises the significance of interventions that promote community engagement, organised postretirement roles and possibilities for ongoing social participation.

### 3.5. Financial Planning

Financial planning emerged as a prominent theme, with teachers emphasising the need for continuous financial education and workshops to create sustainable financial plans for both pre‐retirement and postretirement phases. Participants believed that such education would ensure financial security and increase understanding of savings benefits. A teacher from Jasikan Municipality stated:

*“I seek financial planning education to secure stability in retirement.*” (Participant 11)

Another participant underscored the importance of workshops, noting:

*“I participate in financial planning workshops and seminars to enable me plan for my own retirement*.*”* (Participant 7)

This narrative demonstrates the direct relationship between teachers’ retirement readiness and proactive planning and financial understanding. It highlights the need for institutional programmes that give teachers the needed financial information and tools to enable them to secure their financial security and promote their identity continuity in retirement.

### 3.6. Social and Psychological Planning

The theme of social and psychological planning reflected teachers’ concerns about maintaining a healthy social lifestyle postretirement. Participants expressed a desire for support systems that would reduce isolation and encourage community involvement. One participant articulated:

*“I seek support systems and community activities to reduce isolation after retirement.”* (Participant 13).

*“Personally, I engage in new roles or activities that give meaning to life*”. (Participant 14)

When taken as a whole, these observations highlight the need for institutional and community programmes that combine financial education with chances for identity reinforcement and social interaction. Policymakers can guarantee that retirement becomes a period of stability, dignity and purposeful participation rather than one characterised by insecurity and loneliness by supporting pension literacy classes in addition to leisure activities, community leadership positions and support networks for senior citizens.

## 4. Discussion

A predominant theme emerging from the data was the anxiety surrounding financial concerns for retirement. Many participants expressed fears about their ability to sustain themselves financially once they leave active employment. This concern aligns with existing literature emphasising the importance of financial literacy in shaping positive retirement outcomes [9, 10, 32]. According to Sterns and Spokus [11], many teachers’ worries about their retirement stem from how they will manage their finances after retirement due to inadequate knowledge of pension reimbursement and saving opportunities. These results are consistent with those of Haushofer and Fehr [13], who emphasise how financial constraints impede future‐oriented planning. This is in consistent with Innocenti et al. [12], who posit that people frequently disregard long‐term financial security when faced with present financial concerns. A combination of factors, such as inadequate earnings, low savings capacity and anxiety about pension sufficiency, exacerbates retirement stress. Gerontological models that presume predictable and adequate retirement income systems are challenged by the fact that retirement is not a discrete event but rather the result of long‐term structural disadvantages that accumulate across the life course.

From a stress and coping theory perspective, one of the main ways that retirement is viewed as a threat to well‐being is through financial insecurity. Secondary appraisal processes intended to reduce stress are reflected in the coping mechanisms used by teachers, such as informal savings, reliance on extended family or entrepreneurial endeavours. This approach emphasises how stressors and coping strategies interact to generate retirement‐related worries, which are not just financial but also profoundly psychological. The life‐course perspective also places these concerns in the context of more general trajectories of disadvantage. A combination of factors, such as inadequate earnings, low savings capacity and anxiety about pension sufficiency, exacerbates retirement stress. Gerontological models that presume predictable and adequate retirement income systems are challenged by the fact that retirement is not a discrete event but rather the result of long‐term structural disadvantages that accumulate across the life course.

Healthcare costs have emerged as a significant concern. Anticipated medical expenses contribute to overall anxiety, aligning with research indicating that fears regarding healthcare affordability negatively affect retirement concerns [15]. In Ghana, systemic challenges, such as the ‘cash and carry’ system, exacerbate these anxieties, turning structural deficiencies into individual emotional distress. Stress and coping theory further elucidates how teachers perceive healthcare costs as a significant stressor, while systemic limitations constrain their coping strategies. The life‐course perspective emphasises how prolonged exposure to inadequate healthcare systems contributes to retirement anxieties. This suggests that gerontological models should consider systemic healthcare limitations such as insurance gaps, out‐of‐pocket costs and restricted access that increase stress and hinder effective retirement preparation.

While highlighting shortcomings in current theoretical and policy frameworks, worries over healthcare costs are consistent with gerontological psychology’s emphasis on the critical role of health in successful ageing. These findings demonstrate how expected healthcare expenses and access restrictions influence psychological preparedness for retirement, despite the fact that ageing‐related health decline is generally recognised. This suggests that gerontological models ought to go beyond personal health practices and more clearly take into consideration systemic healthcare limitations, such as insurance coverage gaps and out‐of‐pocket costs, which can worsen stress and compromise the perception of retirement preparation.

The fear of social isolation also emerged as a critical factor influencing retirement concerns. Teachers expressed concerns about losing their social networks and the potential for loneliness in retirement. This aligns with findings that highlight the importance of strong social ties for well‐being during this life stage [16]. For teachers, whose profession is closely linked to their identity and social relationships, retirement signifies a significant disruption. Social identity theory clarifies this disruption: Professional identity and workplace connections are central to self‐concept, and retirement severs these bonds, leading to feelings of disconnection and diminished status [19, 20]. These findings challenge existing beliefs in gerontology that retirees can effortlessly replace workplace networks with connections to family or community. Instead, it underscores the importance of structured interventions such as mentoring programmes, alumni networks and community engagement initiatives that can help maintain a sense of identity and foster social belonging.

Finally, the influence of psychological preparation underscores the proactive role of mental and emotional readiness in shaping retirement concerns. While gerontological literature recognises the importance of psychological adjustment, these findings highlight the significance of anticipatory preparation—specifically, expectations, coping strategies and meaning‐making as essential factors influencing retirement well‐being. Integrating the three frameworks reveals that retirement concerns among basic school teachers in Ghana are multidimensional: Stress and coping theory explains the psychological mechanisms of financial and healthcare anxieties; the life‐course perspective situates these concerns within cumulative trajectories of disadvantage; and social identity theory highlights the relational and identity‐based challenges of retirement. Together, these frameworks demonstrate that effective retirement preparation must integrate financial literacy, systemic healthcare reform and identity continuity programmes, particularly in low‐ and middle‐income contexts where structural vulnerabilities persist.

### 4.1. Limitations and Future Research

Although the study offers insightful information about teachers’ retirement concerns, a number of limitations must be recognised in order to satisfy the rigour expected of a high‐impact qualitative study. First, subjective assessments may have shaped the classification of teachers’ concerns in coding and thematic analysis, where researcher bias may have affected data interpretation. Additionally, the use of self‐reported data raises the possibility of social desirability bias; instead of expressing their true concerns, participants may have modified their answers to seem emotionally stable or financially responsible. This is especially relevant given the study’s findings that many teachers expressed psychological distress about retirement; such admissions may have been underreported due to cultural norms around presenting oneself positively.

Furthermore, limitations in the data collection method, such as reliance on interviews without triangulation from observational or documentary evidence, may have constrained the depth and reliability of the findings. These biases may have influenced the conclusions by underestimating financial insecurity or overestimating coping readiness. Acknowledging these limitations enhances the study’s credibility and emphasises the need for future research to use triangulation, reflexivity and mixed‐methods approaches to reduce bias and capture a more nuanced picture of teachers’ retirement concerns.

Beyond education, future research should study longitudinal studies to follow retirement concerns over time and comparative studies across professions to discover whether these concerns are unique to teachers or reflect broader employee realities in Ghana. Policymakers can ensure that teachers retire with security, dignity and confidence by integrating retirement considerations into career development. This approach will also generate data that can inform changes to national labour policy.

## 5. Conclusion

This study emphasises the importance of pre‐retirement planning and counselling programmes, providing valuable insights into Ghanaian basic school teachers’ views on retirement. The findings indicate that teachers’ concerns are influenced by concerns related to finances, medical expenses and social isolation. These factors emphasise the psychological cost of systemic shortcomings that result in personal emotional suffering by creating a complicated emotional landscape that may have an impact on their general well‐being as they get closer to retirement. The study underscores the need for all‐encompassing support networks that use focused interventions to address these issues. Effective pre‐retirement planning programmes can greatly improve teachers’ retirement concerns, according to the research. These initiatives can reduce anxiety and promote a more optimistic view of retirement by emphasising social connectivity, healthcare understanding and financial literacy.

Preparing teachers for the retirement phase of life requires careful consideration of financial, social and psychological aspects. Overall, the results indicate that enhancing teachers’ views and experiences during this transitional period requires a comprehensive approach to retirement planning. The range of suggestions made by many stakeholders indicates that in order to transition from reactive problem‐solving to proactive, institutionalised support, a national, integrated approach is needed. The study emphasises the importance of holistic retirement planning, which goes beyond financial considerations and places equal focus on mental health and social interactions.

## 6. Recommendations

The study therefore recommends that the Ghana Education Service (GES), the Ghana National Association of Teachers (GNAT), the National Association of Graduate Teachers (NAGRAT) and school heads, in collaboration with financial institutions, should empower teachers with budgeting skills and understanding of pension plans and savings strategies, leading to financial security and reduced anxiety. Additionally, it is suggested that the GES, GNAT, NAGRAT and school heads establish a comprehensive healthcare system in partnership with the National Health Insurance with the goal of meeting teachers’ medical needs at a reasonable cost. Additionally, there have to be well‐planned social media sites that promote interpersonal relationships, offer emotional support, lessen loneliness and promote retirees’ shared experiences.

Furthermore, the study further recommends that in order to encourage teachers to save early, diversify their investments and seek professional advice in order to secure financial security in retirement, the GES, the GNAT, the NAGRAT and school heads should organise financial literacy and planning programmes. In order to give retirees a sense of community, belonging and social stability, the GNAT, the NAGRAT and school heads should cultivate strong peer, family and community networks. To aid with emotional adaptation, pre‐retirement counselling, stress management courses and identity transition workshops should also be offered.

### 6.1. Implications for Policy and Practice

The study revealed that a majority of Ghanaian teachers lack pension knowledge, many are uncertain about healthcare planning, and a significant proportion experience psychological distress regarding life after employment. To enhance stability, dignity and confidence in retirement, our findings highlight the critical need for organised, stakeholder‐led initiatives that combine financial education with psychosocial assistance. The GNAT and the GES should work together to institutionalise retirement education by incorporating it into the framework of continuous professional development (CPD).

Teachers 50 years of age and older should be required to attend yearly workshops addressing important topics such as healthcare planning, budgeting, investment options, entrepreneurship and pension entitlements. Financial professionals and counsellors should lead these meetings to ensure that both technical and psychosocial aspects are covered. School administrators and district and municipal directors of education should spearhead coordination, ensuring fair access across regions and tying attendance to promotion eligibility. Teachers will acquire useful financial skills, a better comprehension of pension systems and increased emotional preparedness for retirement through this comprehensive approach. In the end, these institutionalised programmes will result in greater financial literacy, increased retirement readiness, better healthcare planning and less psychological stress. This will integrate professional development with holistic well‐being and help create a more resilient workforce in education.

### 6.2. Implications for Counselling

Counselling for teachers approaching retirement should extend beyond conventional financial and logistical planning. It needs to embrace a comprehensive approach to well‐being, fostering self‐awareness, cultivating positive relationships and encouraging deliberate participation in meaningful activities. By aligning counselling services with the identified factors influencing teachers’ psychological well‐being, counsellors can better prepare teachers to handle the obstacles of retirement. Financial counselling services are vital for alleviating economic anxiety by providing clear insights into pension planning, effective financial management and sound investment techniques.

Furthermore, the study’s findings indicate the necessity for specialist and regular career seminars, symposia and pre‐retirement planning workshops designed to address the complex problems involved with the pre‐retirement transition. Counselling programmes should focus on giving practical advice and resources for navigating the transition from active teaching to retirement. This includes addressing the redefining of professional identity in the postretirement phase of life, as well as providing strategies for developing and maintaining strong social networks. This approach helps teachers to re‐envision their purpose and reconstruct their professional identity, moving from a potential ‘loss of role’ to finding new avenues for contribution and fulfilment.

## Author Contributions

John N‐yelbi conceived and designed the study, wrote the main manuscript text, prepared figures and also reviewed the manuscript.

Dandy George Dampson contributed to the introduction and methodological framework, supported questionnaire validation and data interpretation, and reviewed and revised the manuscript critically for important intellectual content.

Matthew Kojo Namale reviewed psychometric tools, contributed to the analysis of assessment‐related anxiety data and assisted in cross‐checking data for consistency and integrity.

## Funding

This research was completely self‐funded by the authors. No external grants, sponsorships or institutional financial contributions were obtained. The independence and objectivity of the study were fully upheld throughout the research process.

## Consent

The study did not include any identifiable personal data, multimedia content, or sensitive materials requiring individual publication consent.

## Conflicts of Interest

The authors declare no conflicts of interest.

## Data Availability

The data for the study are available from the corresponding author upon reasonable request.
